# Inferior Outcome of Addition of the Aminopeptidase Inhibitor Tosedostat to Standard Intensive Treatment for Elderly Patients with AML and High Risk MDS

**DOI:** 10.3390/cancers13040672

**Published:** 2021-02-07

**Authors:** Jeroen Janssen, Bob Löwenberg, Markus Manz, Mario Bargetzi, Bart Biemond, Peter von dem Borne, Dimitri Breems, Rolf Brouwer, Yves Chalandon, Dries Deeren, Anna Efthymiou, Bjørn-Tore Gjertsen, Carlos Graux, Michael Gregor, Dominik Heim, Urs Hess, Mels Hoogendoorn, Aurelie Jaspers, Asiong Jie, Mojca Jongen-Lavrencic, Saskia Klein, Marjolein van der Klift, Jürgen Kuball, Danielle van Lammeren-Venema, Marie-Cecile Legdeur, Arjan van de Loosdrecht, Johan Maertens, Marinus van Marwijk Kooy, Ine Moors, Marten Nijziel, Florence van Obbergh, Margriet Oosterveld, Thomas Pabst, Marjolein van der Poel, Harm Sinnige, Olivier Spertini, Wim Terpstra, Lidwine Tick, Walter van der Velden, Marie-Christiane Vekemans, Edo Vellenga, Okke de Weerdt, Peter Westerweel, Georg Stüssi, Yvette van Norden, Gert Ossenkoppele

**Affiliations:** 1 Amsterdam University Medical Centers, loc. VUmc, de Boelelaan 1117, 1081 HV Amsterdam, The Netherlands; a.vandeloosdrecht@amsterdamumc.nl (A.v.d.L.); g.ossenkoppele@amsterdamumc.nl (G.O.); 2 Erasmus Medical Center, 3015 GD Rotterdam, The Netherlands; b.lowenberg@erasmusmc.nl (B.L.); m.lavrencic@erasmusmc.nl (M.J.-L.); 3 Universitätsspital Zürich, 8091 Zürich, Switzerland; markus.manz@usz.ch; 4 Kantonsspital Aarau, 5001 Aarau Aarau, Switzerland; mario.bargetzi@ksa.ch; 5 Amsterdam University Medical Centers, loc. AMC, 1105 AZ Amsterdam, The Netherlands; b.j.biemond@amc.uva.nl; 6 Leiden University Medical Center, 2333 ZA Leiden, The Netherlands; P.A.von_dem_Borne@lumc.nl; 7 Ziekenhuis Netwerk Antwerpen, 2000 Antwerp, Belgium; dimitri.breems@zna.be; 8 Reinier de Graaf Hospital, 2625 AD Delft, The Netherlands; brouwer@rdgg.nl; 9 Division of Hematology, Department of Oncology, Geneva University Hospitals and Faculty of Medicine, University of Geneva, 1211 Geneve, Switzerland; yves.chalandon@hcuge.ch; 10 AZ Delta, 8800 Roeselare, Belgium; dries.deeren@azdelta.be; 11 Hôpital Fribourgeois, 1708 Fribourg, Switzerland; anna.efthymiou@h-fr.ch; 12 Haukeland University Hospital, 5021 Bergen, Norway; bjorn.gjertsen@med.uib.no; 13 Mont Godinne, 5530 Yvoir, Belgium; carlos.graux@uclouvain.be; 14 Luzerner Kantonsspital, 6004 Luzern, Switzerland; michael.gregor@luks.ch; 15 University Hospital of Basel, 4031 Basel, Switzerland; dominik.heim@usb.ch; 16 Kantonsspital St. Gallen, 9007 St.Gallen, Switzerland; urs.hess@kssg.ch; 17 Medisch Centrum Leeuwarden, 8934 AD Leeuwarden, The Netherlands; m.hoogendoorn@znb.nl; 18 Citadelle Hospital, 4000 Liege, Belgium; aurelie.jaspers@chu.ulg.ac.be; 19 Zuyderland MC, Postbus 5500, 6130 MB Sittard, The Netherlands; a.jie@zuyderland.nl; 20 Meander Medical Center, 3813 TZ Amersfoort, The Netherlands; sk.klein@meandermc.nl; 21 Amphia Hospital, 4819 EV Breda, The Netherlands; mvanderklift@amphia.nl; 22 University Medical Center, 3584 CX Utrecht, The Netherlands; j.h.e.kuball@umcutrecht.nl; 23 Haga Hospital, 2545 AA Den Haag, The Netherlands; d.vanlammeren@hagaziekenhuis.nl; 24 Medisch Spectrum Twente, 7512 KZ Enschede, The Netherlands; m.legdeur@mst.nl; 25 University Hospital Gasthuisberg, 3000 Leuven, Belgium; johan.maertens@uz.kuleuven.ac.be; 26 Isala Clinics, 8025 AB Zwolle, The Netherlands; m.van.marwijk@isala.nl; 27 Gent University Hospital, 9000 Gent, Belgium; ine.moors@ugent.be; 28 Catharina Hospital, 5623 EJ Eindhoven, The Netherlands; marten.nijziel@catharinaziekenhuis.nl; 29 Hospital Jolimont, 7100 Haine-Saint-Paul, Belgium; florence.vanobbergh@jolimont.be; 30 Canisius Wilhelmina Hospital, 6532 SZ Nijmegen, The Netherlands; m.oosterveld@cwz.nl; 31 Inselspital, University Hospital, 3010 Bern, Switzerland; thomas.pabst@insel.ch; 32 Maastricht University Medical Center, 6229 HX Maastricht, The Netherlands; marjolein.vander.poel@mumc.nl; 33 Jeroen Bosch Ziekenhuis, 5223 GZ Den Bosch, The Netherlands; H.Sinnige@jbz.nl; 34 Centre Hospitalier Universitaire Vaudois, 1011 Lausanne, Switzerland; Olivier.Spertini@chuv.ch; 35 OLVG, 1091 AC Amsterdam, The Netherlands; w.e.terpstra@olvg.nl; 36 Maxima Medical Center, 5631 BM Eindhoven, The Netherlands; l.tick@mmc.nl; 37 Radboud University Medical Center, 6525 GA Nijmegen, The Netherlands; Walter.vanderVelden@radboudumc.nl; 38 Hopital St. Luc, 1200 Brussels, Belgium; marie-christiane.vekemans@uclouvain.be; 39 University Medical Center, 9713 GZ Groningen, The Netherlands; e.vellenga@umcg.nl; 40 Antonius Hospital, 3435 CM Nieuwegein, The Netherlands; o.weerdt@antoniusziekenhuis.nl; 41 Albert Schweitzer Hospital, 3318 AT Dordrecht, The Netherlands; p.e.westerweel@asz.nl; 42 Istituto Oncologico Della Svizzera Italiana, 6500 Bellinzona, Switzerland; Georg.Stuessi@uzh.ch; 43 Hovon Data Center, Erasmus Medical Center, 3015 GD Rotterdam, The Netherlands; y.vannorden@erasmusmc.nl

**Keywords:** AML, high-risk MDS, tosedostat, clinical trial, aminopeptidase inhibitor, elderly

## Abstract

**Simple Summary:**

Treatment results of acute myeloid leukemia (AML) in elderly patients are unsatisfactory. We investigated in an open label randomized phase II study whether addition of tosedostat, an aminopeptidase inhibitor, to intensive chemotherapy would improve outcome in this population. 231 AML patients > 65 years of age were randomly assigned to receive standard chemotherapy with or without tosedostat for two cycles. We found that complete bone marrow leukemia clearance was not significantly different between both arms. After two years, survival was 33% for the standard arm versus 18% for the tosedostat arm. More patients died due to infectious complications in the tosedostat arm than after standard treatment. Also, a cardiac rhythm abnormality called atrial fibrillation was more often seen in the tosedostat arm. We conclude that the addition of tosedostat to standard chemotherapy does negatively affect the therapeutic outcome of elderly patients with acute myeloid leukemia.

**Abstract:**

Treatment results of AML in elderly patients are unsatisfactory. We hypothesized that addition of tosedostat, an aminopeptidase inhibitor, to intensive chemotherapy may improve outcome in this population. After establishing a safe dose in a run-in phase of the study in 22 patients, 231 eligible patients with AML above 65 years of age (median 70, range 66–81) were randomly assigned in this open label randomized Phase II study to receive standard chemotherapy (3+7) with or without tosedostat at the selected daily dose of 120 mg (*n* = 116), days 1–21. In the second cycle, patients received cytarabine 1000 mg/m^2^ twice daily on days 1-6 with or without tosedostat. CR/CRi rates in the 2 arms were not significantly different (69% (95% C.I. 60–77%) vs 64% (55–73%), respectively). At 24 months, event-free survival (EFS) was 20% for the standard arm versus 12% for the tosedostat arm (Cox-p = 0.01) and overall survival (OS) 33% vs 18% respectively (*p* = 0.006). Infectious complications accounted for an increased early death rate in the tosedostat arm. Atrial fibrillation was more common in the tosedostat arm as well. The results of the present study show that the addition of tosedostat to standard chemotherapy does negatively affect the therapeutic outcome of elderly AML patients.

## 1. Introduction

Acute myeloid leukemia is primarily a disease of the elderly. In these patients the disease has an even worse perspective than in younger patients because of poor tolerance to induction chemotherapy treatment and specific disease characteristics, with overrepresentation of poor risk cytogenetics and molecular abnormalities. For fit elderly patients, intensive chemotherapy with idarubicin/daunorubicin and cytarabine results in complete remissions in 60–70% of patients, but only 20–25% of patients will have long term leukemia-free survival due to a high rate of relapse.

Clearly, these results need to be improved. Unfortunately, very little progress has been made to date. The addition of gemtuzumab ozogamycin results in better outcomes but only in the good-risk and intermediate-risk category and particularly in the core binding factor leukemias that are rare in the elderly [1]. Exchange of standard anthracycline/cytarabine combinations with a liposomal formulation (CPX-351) also leads to improved outcomes with reduced toxicity, but its use is only approved for secondary AMLs and those with myelodysplasia related changes [2]. In addition, increased use of allogeneic stem cell transplantation in the elderly age groups has also resulted in modest improvements of treatment results [3].

While the benefit of new targeted compounds like venetoclax and IDH1/2 inhibitors as addition to hypomethylating agents has already been demonstrated in palliative treatment of unfit patients, their role in potentially curative intensive regimens for fit patients is still unclear [4,5,6].

In an effort to improve the outcome of fit elderly AML patients, the HOVON/SAKK collaborative group designed the HOVON 103 study where several promising compounds were added to the standard 3+7 backbone in a so-called Octopus design. Every compound was tested in a randomized way against the standard treatment in relatively small groups of patients, in order to rapidly select an active drug that would have a large impact on complete remission rates. With this design, around 100 patients per experimental arm would be needed. Results of the addition of lenalidomide have recently been published [7]. Here, we report on the results of the tosedostat arm. Tosedostat, the abbreviated name of the oral ester moiety 2S-[2R-(S-hydroxy-hydroxycarbamoyl-methyl)-4-methylpentanoylamino]-2-phenylethanoic acid, is a cyclopentyl ester which has aminopeptidase inhibitory activity. Inhibition of aminopeptidase leads to a reduction of protein recycling by a deprivation of free amino acids in the cell and secondarily to an amino acid deprivation response, ultimately resulting in a reduction of protein synthesis and cell proliferation [8]. In rapidly dividing cells like AML cells, this leads to apoptosis. The drug has shown promising activity in Phase I studies and shows synergism with cytarabine in AML cell lines [9]. It was therefore a logical step to test the drug in combination with intensive chemotherapy regimens in AML patients.

Following a dose feasibility run-in study, we selected a dose level of 120 mg tosedostat for the definitive Phase II randomized study. Here, we report the results of the addition of tosedostat to standard 3+7 treatment in a prospective Phase 2 randomized study of 231 patients.

## 2. Results

**Figure 1:**
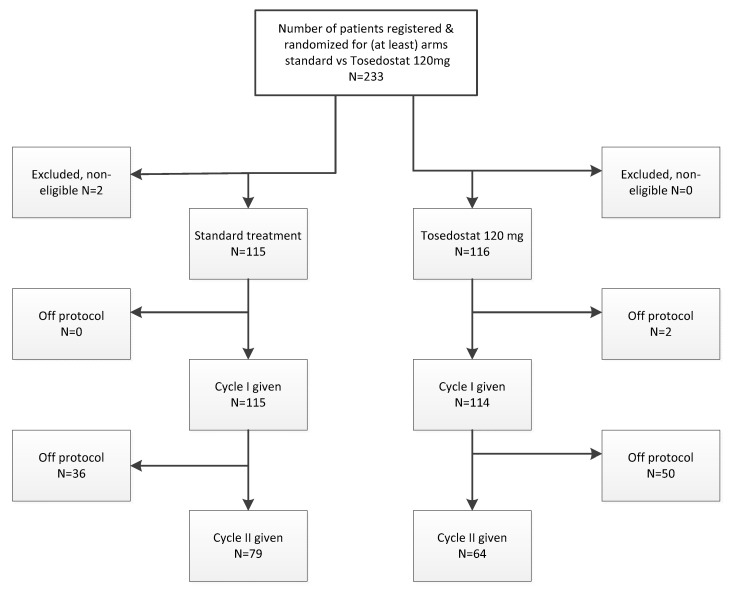
Consort diagram of the study. Note: the twenty-three patients that have been treated at tosedostat 180 mg qd have been excluded from this figure.

The tosedostat arm of the study was activated in 2010 and closed after completion of accrual in 2016. Based on FDA recommendations based on the number of dose limiting toxicities in a then ongoing phase 1 study, after 23 patients had been treated at tosedostat 180 mg qd, the dose which had been active after first interim analysis, all further enrolled patients were treated with 120 mg qd. Median FU of patients still alive is 29 months. The analysis presented here include 116 patients treated on the tosedostat 120 mg arm and 115 patients in the control arm receiving standard treatment. See CONSORT diagram shown in Figure 1.

### 2.1. Patients

Patient characteristics at diagnosis by treatment arm are shown in Table 1. Median age of the patients was less than one year higher in the experimental arm: 70 versus 69 years with slightly more patients being >70 years of age. Other major known risk factors were well-balanced over both arms.

### 2.2. Treatment, Response, and Outcome

Of 231 eligible patients, 229 patients received the first treatment cycle and 223 (97%) received full doses of daunomycin according to the protocol and 222 (97%) received full doses of cytarabine in cycle 1. Sixty-six patients out of 114 (58%) completed the full series of doses of tosedostat in cycle 1. The majority of the patients who did not receive the protocol-specified dosages of tosedostat discontinued early due to toxicity. Length of stay in the hospital was on average two days longer in the tosedostat arm than in the standard arm (median 30 days compared to 28 days).

In cycle 2, cytarabine could be administered at full dose in 74 of 79 patients (94%) in the standard arm and in 62/64 (97%) of the experimental arm. Tosedostat was given according to the protocol in only 19 of 64 patients (30%), with 30/64 patients (47%) stopped early and, as in cycle I, dose modifications were mainly due to toxicity. Nineteen patients (17%) in the standard arm and 10 (9%) in the experimental arm received an upfront alloHSCT.

**Figure 2:**
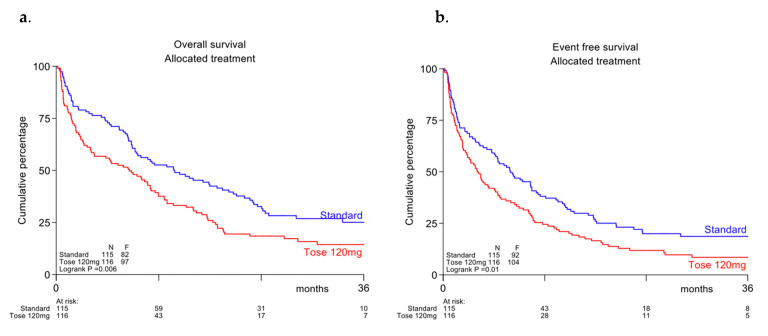
(**a**) Overall survival (**b**) Event-free survival.

CR/CRi rate on induction in the tosedostat arm was 64% (95%-CI: 55–73%) and 69% (95%-CI: 60–77%) in the control arm (*p* = NS). With a median follow-up time of patients still alive of 29 months, the overall survival in the tosedostat arm was significantly lower than in the control arm (Cox-*p* = 0.006, overall survival (OS) at 2 years 18% vs. 33%, see Figure 2a), as was event-free survival (*p* = 0.01; EFS at 2 yrs 12% versus 20, see Figure 2b) and disease-free survival (*p* = 0.02; DFS at 2 y 17% vs. 28%, not shown). Due to the limited number of patients, no separate survival analyses were done for the individual molecular subgroups. 

Early death rates were higher in the tosedostat arm than in the standard arm. Within 30 days, 19% of patients had died in the experimental arm, compared to 8% in the standard arm. The 60 days death rates were 28% versus 19%, respectively. See Table 2 for an overview of these results.

### 2.3. Adverse Events and Hematological Recovery

In Tables S1 and S2, the number of AEs in cycles 1 and 2 by diagnosis category, common toxicity criteria (CTCAE version 4) grade, and arm of randomization are given. The frequencies of grade 3 and 4 CTCs appear higher in the tosedostat arm, with especially an increased occurrence of infectious grade 4 AEs (22% vs. 5%) during cycle 1. This was paralleled by the increased early death rate with 13 of 22 deaths within 30 days in the tosedostat arm due to infectious causes, whereas this was only 2 of 9 in the standard arm. This was also evident in cycle 2. 

Remarkably, an increased number of cases with atrial fibrillation was seen in the tosedostat arm (18% in cycle 1, versus 4% in the control arm, 9% and 5%, respectively, in cycle 2, see Table S4), whereas other AEs were comparable between both treatment arms.

**Figure 3:**
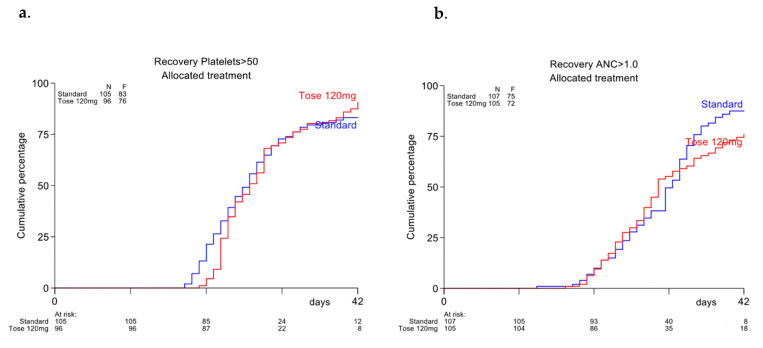
(**a**) Recovery time after cycle 1 to absolute neutrophil count > 1.0 × 10^9^/L (**b**) Recovery time after cycle 1 to platelets > 50 × 10^9^/L.

Time to neutrophil or platelet recovery between the two groups did not significantly differ after cycle 1 nor after cycle 2 (see Figure 3).

### 2.4. Measurable Residual Disease (MRD)

In 69 patients (36 in the standard arm and 33 in the experimental arm) MRD was assessed by multiparameter flow cytometry. In the control arm 75% became MRD negative as compared to 67% in the tosedostat arm. OS at 2 y was 54% for the MRD negative patients and 18% for the MRD positive patients (*p* < 0.001) (see Figure S1). Disease-free survival at two years was 32% and 7%, respectively (*p* = 0.005).

## 3. Discussion

In this open label randomized Phase II trial, we treated fit patients of older age with intensive chemotherapy with or without the addition of tosedostat, an aminopeptidase inhibitor that had shown promising results in several previous smaller trials, where the drug had either been used as a single agent or in combination with less intensive chemotherapy [9,10,11,12,13]. Exposure to tosedostat of leukemic blasts in vitro leads to an amino acid deprivation response, and ultimately to apoptosis. There was a strong synergistic effect with cytarabine [8]. Tosedostat shuts down the cellular protein recycling machinery by blocking degradation of proteasome processed proteins to amino-acids. Remarkably, mammalian cells appear strongly dependent on theses recycled amino acids for their protein synthesis, even in an extracellular environment where amino acids are abundantly available. Rapidly dividing cells like leukemic blasts seem especially dependent on this mechanism and thereby, the drug is expected to show moderate leukemia-specificity.

The HOVON 103 study was designed to test multiple new promising drugs in a randomized Phase II setting. In this way, first, lenalidomide was tested and showed no improvement in outcome as recently reported [7]. The results of the current tosedostat study are quite disappointing. Although CR rates were comparable between the two arms, reduced survival was seen in the tosedostat arm. This appears to be caused by increased toxicity related to the addition of the experimental drug. While bone marrow recovery was not significantly delayed by tosedostat, a higher incidence of (fatal, and mostly bacterial) infections was seen. 

Previous studies with tosedostat as a single agent in relapsed/refractory AML cases did not show a signal of increased infection propensity [9,11]. However, in single arm studies where the drug was combined with intermediate dose cytarabine, decitabine or low dose cytarabine, rates of grade 3–4 infectious complications were up to 47% [12]. As the drug did not induce slower recovery of blood cell counts in our study, the higher infection rate must be explained otherwise. In this regard, it is of interest that aminopeptidase inhibition shuts down production of pro-inflammatory cytokines by monocytes whereas it increases synthesis of anti-inflammatory cytokines [14]. Tosedostat also inhibits CD13, (an aminopeptidase itself), which leads to reduced phagocytic activity of macrophages and to reduced development and maturation of dendritic cells from monocytes [14]. In addition, tosedostat, through its intracellular amino acid deprivation activity displays mTOR inhibitory activity, which may limit T-cell proliferation and T-cell differentiation towards effector T-cells [15]. The drug may also reduce peptide processing for HLA presentation [8] A significant subset of T-cells displays esterase activity and may thus intracellularly capture the active tosedostat metabolite [16]. The severe infections that were seen in the tosedostat arm were mainly of bacterial origin. Fungal infections were not increased compared to the control arm, which may relate to the antifungal activity that tosedostat also exposes [17].

Apparently, addition of new drugs to the current 3+7 standard of chemotherapy proves to be a difficult developmental path to pursue. With many compounds having been tested by HOVON and other groups, it is discouraging to see the accumulating disappointing results of these efforts. It appears that elderly patients can hardly tolerate any additional toxicity from additive cytotoxic agents when added to the already toxic 3+7 treatment schedule.

Recently, several new and promising targeted compounds have become available, for treatment of elderly patients with or without specific molecular characteristics, like IDH1 and -2 inhibitors, FLT3- inhibitors and the bcl-2 inhibitor venetoclax and these have in part, as adjunct to low dose cytarabine or hypomethylating agents, proven beneficial in the unfit patient groups [18,19]. Hopefully, as these new agents have a rather beneficial toxicity profile, they will also prove valuable as an addition to more intensive and possibly curative chemotherapy. New immunotherapeutic strategies are also appearing on the horizon, but their efficacy still needs to be proven.

## 4. Materials and Methods

### 4.1. Patients

Previously untreated patients, 66 years of age or older, with a cytologically confirmed diagnosis of de novo or secondary AML (not acute promyelocytic leukemia or CML blast crisis) or with refractory anemia with excess of blasts and a Revised International Prognostic Scoring System (IPSS) score of higher than 4.5 and a WHO performance score of 2 or less were eligible for inclusion. Except for hydroxyurea for <2 weeks, no other previous AML treatment was allowed. Exclusion criteria included clinically significant cardiovascular disease, including cerebrovascular accidents (<6 months before randomization), myocardial infarction (<6 months before randomization), unstable angina, New York Heart Association grade 2 or greater congestive heart failure, serious cardiac arrhythmia requiring medication and other standard general medical exclusions. The trial was approved by the institutional review boards of all participating institutions. The study was performed in accordance with the Declaration of Helsinki, and all patients provided written informed consent.

### 4.2. Risk Classification

Based on clinical characteristics, karyotype, and molecular genotype of the leukemic cells, patients were classified into prognostic categories according to Table S1.

### 4.3. Study Design and Chemotherapy

Tosedostat was provided free of charge by Chroma Therapeutics (Abingdon, United Kingdom). The study was divided in two parts. The first part was planned to be a randomized dose selection run-in phase with oral tosedostat 120 mg/day 1–21 in cycle 1 and day 1–56 in cycle 2 added to standard induction chemotherapy. Escalation to 180 mg and 240 mg/day was initially planned to be decided upon evaluation of the toxicity profiles after each dose level, but after 22 patients were enrolled at the 180 mg level, upon FDA recommendation, the dose was again reduced to 120 mg for the ensuing open label Phase II part. 

During the Phase II part, one interim analysis regarding efficacy was performed after enrollment of 100 patients (50 per arm) on the primary endpoint according to protocol. Patients were randomly assigned to remission induction regimens with or without tosedostat. Cycle 1 consisted of daunorubicin at 45 mg/m^2^ (3-hr infusion on days 1, 2 and 3) and cytarabine at a dose of 200 mg/m^2^ (per continuous infusion on days 1–7) with or without tosedostat at 120 mg, days 1–21. Cycle 2 contained cytarabine 1000 mg/m^2^ q 12 hrs via 6 hrs infusion from day 1–6 (12 doses) with or without tosedostat at 120 mg/day 1–56. Tosedostat was stopped when after day 35, platelets were still <30 × 10^9^/L and/or ANC < 0.5 × 10^9^/L. Patients could be allotransplanted off protocol according to local policy. MRD analysis and detection was performed as previously described [20].

### 4.4. Statistical Analysis

The primary endpoint of the second part of the study was the rate of complete remission after induction treatment. A patient was considered to have a response if the best response to remission induction therapy (cycle 1 and/or 2) was a CR/CRi. Secondary endpoints were considered as exploratory and included: overall survival (OS), event free survival (EFS), disease free survival (DFS), the prognostic value of leukemic molecular markers and gene expression profiles and the prognostic value of minimal residual disease measurements following therapy. The definitions which are standard are according the ELN recommendations [21]. A planned futility interim analysis was incorporated after 100 patients were randomized.

At final analysis, tosedostat was considered not effective as addition to standard chemotherapy if no difference in CR/CRi rate in favor of tosedostat was seen, i.e., when the upper limit of the 80% confidence interval (CI) of the difference in CR rate would be ≤15%, which was the case if the observed difference in complete response rate was less than 2% in favor of the tosedostat arm. Otherwise, we would consider to continue as Phase III. Kaplan–Meier survival curves and Cox tests were used to compare the survival distributions between the treatment arms.

## 5. Conclusions

In this prospective randomized Phase II study, addition of tosedostat to the current 3+7 standard of chemotherapy did not improve outcome in elderly AML patients. In fact, results were inferior in the experimental arm, due to more infection-related deaths.

## Figures and Tables

**Table 1:** Baseline patient characteristics.

	Standard Arm (*n* = 115)	Tosedostat 120 mg (*n* = 116)	Total
Sex			
M	72 (63%)	76 (66%)	148 (64%)
F	43 (37%)	40 (34%)	83 (36%)
Age groups			
≤70 years	72 (63%)	64 (55%)	136 (59%)
>70 years	43 (37%)	52 (45%)	95 (41%)
Age			
Mean; SD	69.9; 3.26	70.7; 3.75	70.3; 3.53
Median; range	69; 66–79	70; 66–81	70; 66–81
WHO performance			
0	63 (55%)	54 (47%)	117 (51%)
1	43 (37%)	50 (43%)	93 (40%)
2	8 (7%)	11 (9%)	19 (8%)
NA	1 (1%)	1 (1%)	2 (1%)
Diagnosis			
MDS	15 (13%)	16 (14%)	31 (13%)
AML	100 (87%)	100 (86%)	200 (87%)
Prior HM			
No	109 (95%)	106 (93%)	215 (94%)
Yes	6 (5%)	8 (7%)	14 (6%)
AML risk group (acc. to HOVON 103 protocol) *			
Good	8 (7%)	2 (2%)	10 (4%)
Intermediate	35 (30%)	39 (34%)	74 (32%)
Poor	60 (52%)	57 (49%)	117 (51%)
Very poor	12 (10%)	18 (16%)	30 (13%)
*NPM1* mutation			
Neg	51 (44%)	53 (46%)	104 (45%)
Pos	19 (17%)	19 (16%)	38 (16%)
NA	45 (39%)	44 (38%)	89 (39%)
*FLT3ITD*			
Neg	60 (52%)	67 (58%)	127 (55%)
Pos	12 (10%)	7 (6%)	19 (8%)
NA	43 (37%)	42 (36%)	85 (37%)
*FLT3 TKD835*			
Neg	33 (29%)	31 (27%)	64 (28%)
Pos	2 (2%)	3 (3%)	5 (2%)
NA	80 (70%)	82 (71%)	162 (70%)
*EVI1* overexpression			
Neg	65 (57%)	61 (53%)	126 (55%)
Pos	8 (7%)	11 (9%)	19 (8%)
NA	42 (37%)	44 (38%)	86 (37%)
*CEBPA DM*			
Neg	63 (55%)	68 (59%)	131 (57%)
Pos	4 (3%)	2 (2%)	6 (3%)
NA	48 (42%)	46 (40%)	94 (41%)
*FLT3ITD × NPM1* mutation			
Pos × Pos	9 (8%)	5 (4%)	14 (6%)
Pos × neg	3 (3%)	1 (1%)	4 (2%)
Neg × Pos	10 (9%)	14 (12%)	24 (10%)
Neg × Neg	47 (41%)	50 (43%)	97 (42%)
NA	46 (40%)	46 (40%)	92 (40%)

HM: Hematological Malignancy; NA: Not available; * see Table S1.

**Table 2:** Treatment outcome of patients randomized to standard chemotherapy with or without tosedostat.

	Standard Treatment	Tosedostat 120 mg	HR (95%-C.I.)	*p*
Complete remission (CR/CRi) (95% C.I.)	69% (60–77)	64% (55–73)		0.431
CR/CRi (after cycle I)	54%	56%		
PR (after cycle 1)	4%	5%		
RD (after cycle 1)	24%	18%		
PR (after cycle 2)	0%	0%		
RD (after cycle 2)	6%	6%		
Death within 30 days	8%	19%		
Death within 60 days	19%	28%		
OS at 2 years	33	18	1.51 (1.12–2.03)	0.006
EFS at 2 years	20	12	1.44 (1.08–1.90)	0.01
DFS at 2 years	28	17	1.51 (1.06–2.16)	0.02

CRi: Complete remission with incomplete hematological recovery; C.I.: Confidence interval; HR: Hazard ratio; PR: Partial remission; RD: Refractory disease; OS: overall survival; EFS: event-free survival; DFS: disease-free survival.

## Data Availability

The data presented in this study are available on request from the corresponding author. The data are not publicly available due to pending HOVON data sharing policy.

## Supplementary Materials

The following are available online at https://www.mdpi.com/2072-6694/13/4/672/s1, Figure S1: Overall survival (OS) (A) and disease free survival (DFS) (B) for MRD negative or positive patients; Table S1: Risk group classification as used in HOVON 103 protocol; Table S2: Adverse events in Cycle 1; Table S3: Adverse events in Cycle 2; Table S4: Atrial fibrillation in period 1 and 2, per arm.
