# Supplementary material for: Inferior Outcome of Addition of the Aminopeptidase Inhibitor Tosedostat to Standard Intensive Treatment for Elderly Patients with AML and High Risk MDS
Source: Cancers (Basel). 2021 Feb 7;13(4):672. doi: 10.3390/cancers13040672 (PMC7914531; doi:10.3390/cancers13040672)
Supplement: Supplementary file 1 [file cancers-13-00672-s001.pdf]

Table S1. Risk group classification as used in HOVON 103 protocol.

Risk	Definition
Good	t(8;21) or AML1-ETO, WBC≤20 x 10^9/L
	inv(16)/t(16;16) or CBFB-MYH11 gene
	MK-, CEBPA+
	MK-, FLT3ITD-/NPM1+, CRe
Intermediate	t(8;21) or AML1-ETO, WBC>20 x 10^9/L
	CN –X –Y, WBC≤100 x 10^9/L, CRe
Poor	CN –X –Y, WBC≤100 x 10^9/L, not CRe
	CN –X –Y, WBC>100 x 10^9/L
	CA, non CBF, MK-, no abn3q26, EVI1-
Very Poor	Non-CBF, MK+
	Non-CBF, abn3q26
	Non-CBF, EVI1+

WBC: White blood cells; CRe: Early CR, after cycle 1. MK: Monosomal karyotype; CN: Normal karyotype; CA: Chromosomal abnormalities; CBF: Core-binding factor.

Table S2. Adverse events and maximum grade in period 1, per arm.

**Arm**	**Standard arm (n=115)**						**Tosedostat 120mg arm (n=114)**					
**CTC grade**	**2**		**3**		**4**		**2**		**3**		**4**	
	**n**	**%**	n	%	n	%	n	%	n	%	n	%
Infection and infestations	28	24%	28	24%	6	5%	21	18%	31	27%	25	22%
Gastrointestinal disorders	31	27%	15	13%	2	2%	33	29%	24	21%	3	3%
Blood and lymphatic system disorders	3	3%	51	44%	2	2%	1	1%	43	38%	1	1%
Metabolism and nutrition disorders	11	10%	20	17%	.	.	13	11%	28	25%	3	3%
Skin &subcutaneous tissue disorders	28	24%	8	7%	.	.	30	26%	7	6%	.	.
Investigations	9	8%	11	10%	3	3%	22	19%	9	8%	5	4%
Respiratory thoracic& mediastinal dis	11	10%	8	7%	8	7%	11	10%	5	4%	13	11%
Gen. disorders & admin. site conditions	21	18%	4	3%	2	2%	22	19%	6	5%	.	.
Cardiac disorders	3	3%	6	5%	4	3%	23	20%	8	7%	4	4%
Vascular disorders	14	12%	5	4%	2	2%	10	9%	3	3%	2	2%
Nervous system disorders	5	4%	4	3%	2	2%	8	7%	4	4%	.	.
Psychiatric disorders	7	6%	1	1%	.	.	7	6%	1	1%	.	.
Renal and urinary disorders	4	3%	2	2%	.	.	6	5%	3	3%	1	1%
Immune system disorders	1	1%	1	1%	.	.	6	5%	1	1%	.	.
Eye disorders	5	4%	.	.	.	.	3	3%	.	.	.	.
Ear and labyrinth disorders	5	4%	.	.		.	1	1%	1	1%		
Musculoskeletal& connective tissue dis	5	4%	.	.	.	.	1	1%	.	.	.	.
Injury poisoning& procedural complic	3	3%	.	.	.	.	1	1%	1	1%	.	.
Hepatobiliary disorders	.	.	.	.	.	.	.	.	1	1%	1	1%
Reproductive system & breast disorders	1	1%	.	.	.	.	.	.	.	.	1	1%
Endocrine disorders	.	.	1	1%.	.	.	.	.	.	.	.	.
Surgical and medical procedures	1	1%	.	.	.	.	.	.	.	.	.	.

Table S3. Adverse events and maximum grade in period 2, per arm.

**Arm**	**Standard (n=79)**	**Tosedostat 120 mg (n=64)**

CTC grade	2		3		4		2		3		4	
	n	%	n	%	n	%	n	%	n	%	n	%
Gastrointestinal disorders	35	44%	13	16%	2	3%	24	38%	11	17%	.	
Infections and infestations	19	24%	21	27%	5	6%	9	14%	22	34%	9	14%
Skin & subcutaneous tissue disorders	29	37%	9	11%	.		24	38%	2	3%		
Blood and lymphatic system disorders	5	6%	23	29%	2	3%	1	2%	21	33%	.	
Metabolism and nutrition disorders	10	13%	19	24%	.	.	4	6%	17	27%	.	.
Investigations	3	4%	12	15%	.	.	8	13%	5	8%	2	3%
Cardiac disorders	6	8%	1	1%	.	.	10	16%	3	5%	2	3%
Gen. disorders & admin. site conditions	5	6%	3	4%	1	1%	8	13%	2	3%	3	5%
Respiratory thoracic & mediastinal dis.	4	5%	2	3%	1	1%	6	9%	1	2%	4	6%
Nervous system disorders	7	9%	1	1%	.	.	5	8%	2	3%	1	2%
Vascular disorders	5	6%	3	4%	.	.	4	6%	3	5%	.	.
Eye disorders	5	6%	1	1%	.	.	4	6%	2	3%	.	.
Renal and urinary disorders	4	5%	2	3%	.	.	3	5%	1	2%	1	2%
Psychiatric disorders	5	6%	.	.	.	.	5	8%	.	.	.	.
Musculoskeletal & connective tissue dis.	4	5%	.	.	.	.	2	3%	1	2%	.	.
Hepatobiliary disorders	3	4%	.	.	1	1%	.	.	1	2%	.	.
Immune system disorders	2	3%	.	.	.	.	3	5%	.	.	.	.
Injury poisoning & procedural complic.	1	1%	1	1%	.	.	.	.	2	3%	.	.
Neoplasms benign malignant &unspecified	4	5%	.	.	.	.	.	.	.	.	.	.

Table S4. Atrial fibrillation in period 1 and 2, per arm.

	Standard		Tosedostat 120mg	
**Cycle I**				
	n	%	n	%
No	110	96%	94	82%
Yes	5	4%	20	18%
Cycle II				
No	75	95%	58	91%
Yes	4	5%	6	9%

a.

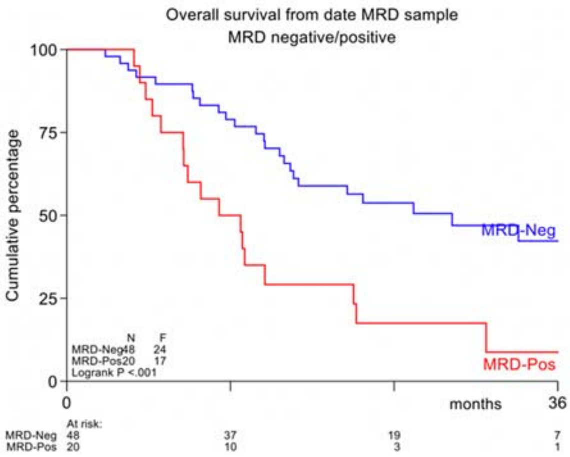

b.

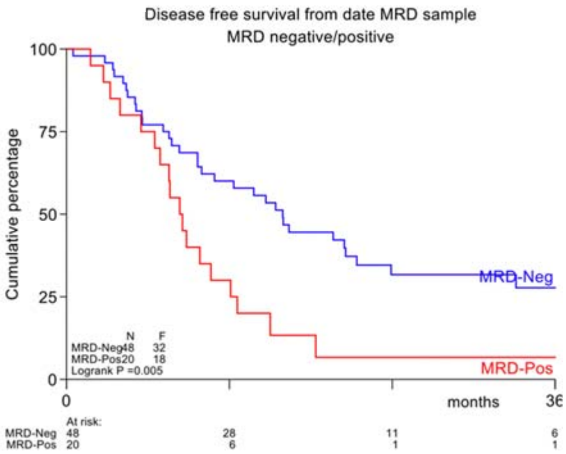

Figure S1. Overall survival according to MRD status after cycle 2. **b.** Disease-free survival according to MRD status after cycle 2.
